# Wild genius - domestic fool? Spatial learning abilities of wild and domestic guinea pigs

**DOI:** 10.1186/1742-9994-7-9

**Published:** 2010-03-25

**Authors:** Lars Lewejohann, Thorsten Pickel, Norbert Sachser, Sylvia Kaiser

**Affiliations:** 1 Department of Behavioural Biology, University of Münster, 48149 Münster, Germany; 2 Otto Creutzfeldt Center for Cognitive and Behavioral Neuroscience, University of Münster, Germany

## Abstract

**Background:**

Domestic animals and their wild relatives differ in a wide variety of aspects. The process of domestication of the domestic guinea pig (*Cavia aperea *f. *porcellus*), starting at least 4500 years ago, led to changes in the anatomy, physiology, and behaviour compared with their wild relative, the wild cavy, *Cavia aperea*. Although domestic guinea pigs are widely used as a laboratory animal, learning and memory capabilities are often disregarded as being very scarce. Even less is known about learning and memory of wild cavies. In this regard, one striking domestic trait is a reduction in relative brain size, which in the domesticated form of the guinea pig amounts to 13%. However, the common belief, that such a reduction of brain size in the course of domestication of different species is accomplished by less learning capabilities is not at all very well established in the literature. Indeed, domestic animals might also even outperform their wild conspecifics taking advantage of their adaptation to a man-made environment.

In our study we compared the spatial learning abilities of wild and domestic guinea pigs. We expected that the two forms are different regarding their learning performance possibly related to the process of domestication. Therefore wild cavies as well as domestic guinea pigs of both sexes, aged 35 to 45 days, were tested in the Morris water maze to investigate their ability of spatial learning.

**Results:**

Both, wild cavies and domestic guinea pigs were able to learn the task, proving the water maze to be a suitable test also for wild cavies. Regarding the speed of learning, male as well as female domestic guinea pigs outperformed their wild conspecifics significantly. Interestingly, only domestic guinea pigs showed a significant spatial association of the platform position, while other effective search strategies were used by wild cavies.

**Conclusion:**

The results demonstrate that domestic guinea pigs do not at all perform worse than their wild relatives in tests of spatial learning abilities. Yet, the contrary seems to be true. Hence, artificial selection and breeding did not lead to a cognitive decline but rather to an adaptation to man-made environment that allows solving the task more efficiently.

## Background

The process of domestication led to significant changes of characteristics that are still to be found in the wild ancestral species. The amount of changes in morphology, physiology, and behaviour may depend upon different adaptations to captivity as well as upon the motivations and purposes the domesticated species was segregated from its origin population and bred for [1-3]. Many domesticated species differ very conspicuously from their ancestors (e.g., poodles from wolves) but others may not (e.g., wild from domestic rats). Regardless of the amount of change, domestication led to intraspecific changes only but did not bring about the origin of a new species [4-6]. A general phenomenon of domestication is a reduction in brain size [6-8]. This reduction has been found in all investigated species with the exception of *Mus musculus *[6]. Noteworthy, comparable allometric dependencies between body- and brain weight are true within groups of wild ancestral species as well as for their domestic forms, thus the reduced brain size of domestic animals is not the result of an increased body weight [6]. Although it is disputable whether or not, and to what extend, brain size matters [9-12], the reduction of relative brain size during domestication is often thought to be reflected in a reduction of functional capacities [6,13]. However, although it is a common believe that domestication reduces the cognitive abilities of a species, experimental evidence supporting this theory is scarce. Contrary, there is evidence for some domestic species to out-compete their wild ancestral forms especially in tasks comprising social skills [14,15].

Guinea pigs (*Cavia aperea *f. *porcellus*) were domesticated at least 4.500 years ago [16] in the highlands of South America providing the Indians with meat and sacrificial animals. In the 16^th ^century domestic guinea pigs were brought to Europe where they were subjected to further selective breeding leading to the common domestic form that is nowadays used as pets and laboratory animals [17]. Their wild relative, the wild cavy (*Cavia aperea*) still is one of the most common and widespread rodents of South America [18-20]. Their natural habitat consists of open areas used for short feeding periods as well as of covered zones of dense vegetation, pervaded by a complex network of runways [18]. Certainly, such structures demand skilled spatial memory and thus considerable spatial memory capabilities were expected for the domestic guinea pig, too [21]. As male wild cavies obtain considerable larger home ranges it is hypothesized that this might be reflected in improved spatial memory of males [22].

Since more than a hundred years, different attempts to analyze learning and memory in guinea pigs have been made. Although guinea pigs were able to learn simple labyrinths [23] and to discriminate between different stimuli [24-26], they did not prove to be the best suited species for this kind of tasks [24]. Thus, most contemporary studies on learning and memory in rodents are conducted with rats or mice [27]. More recently Beck et al. [28] consider guinea pigs even to be a suitable model for the study of Alzheimer's disease with regard to the processing of amyloid precursor protein, but they also conclude that "guinea pigs are not a proper animal species to perform learning or memory tasks". In contrast to this view others [21,29,30] have reported spatial learning in guinea pigs using the Morris water maze task. This task is very commonly used with rats and mice for many years to assess associative, spatial learning. The objection of the test is to find an escape platform that is hidden below the water surface within a circular water pool surrounded by distinct spatial cues [31,32]. Domestic guinea pigs were found to be skilled swimmers and reliably learned the task [21,29,30,33,34].

Wild cavies differ in many aspects from domestic guinea pigs. Domestication of the cavy led to reduced aggressiveness, increased sociopositive behaviour, more male courtship behaviour, and reduced stress reactivity [35,36]. However, concerning their memory skills, not much is known about wild cavies. To our knowledge, there is only a single case study of one wild cavy performing less good than domestic guinea pigs in a discrimination task [25]. In taking advantage of the newly described procedure to analyze spatial memory in guinea pigs using the Morris water maze, the aim of this study is to compare wild cavies and domestic guinea pigs in this regard.

## Methods

### Subjects

Experimental subjects were wild cavies (*Cavia aperea*) and domestic guinea pigs (*Cavia aperea *f. *porcellus*) of both sexes. Overall, 15 male domestic guinea pigs (DM), 13 female domestic guinea pigs (DF), 13 male wild cavies (WM), and 13 female wild cavies (WF) were included. Domestic guinea pigs were descendents of a heterogeneous shorthaired and multicoloured stock of 40 animals obtained from a breeder in 1975 regularly restocked by unrelated guinea pigs from local breeders. These animals could be individually identified by natural markings. The wild cavies derived from animals trapped in the wild in the Province of Buenos Aires, Argentina, in 1974 and 1995. In 2003 a study conducted in our department indicated that the long-term breeding and rearing of wild guinea pigs in captivity did not result in significant changes in behaviour and hormonal stress responses [36]. Wild cavies were marked by bleaching parts of their fur with hydrogen peroxide.

### Housing conditions and maintenance

Animal maintenance was under standardized conditions, with a 12:12 light-dark-cycle and a photoperiod from 07:00 to 19:00 h. The room temperature was maintained at 23 ± 2°C, and relative humidity was about 50%. All animals were offspring of laboratory-reared pair-housed individuals. For practical reasons, enclosure sizes of the breeding pairs of wild cavies were 1.0 - 1.5 m^2^, while the rearing enclosures of domestic guinea pigs were 0.5 m^2^. After weaning at an age of 21 days all animals were housed in unisex groups of two to five individuals in enclosures of the same size measuring 1.5 m^2^. Enclosures were cleaned weekly and provided with fresh bedding (Allspan, Karlsruhe, Germany). Commercial guinea pig diet (Höveler "Spezialfutter" 10700, Höveler Spezialfutterwerke GmbH & Co. KG Dormagen, Germany; Altromin 3023, Altromin GmbH Lage, Germany), oat flakes, and vitamin C fortified tap water were available *ad libitum*. The diet was regularly supplemented with hay and straw. During the testing procedure, animals were weighed daily in order to assure their well-being. All experiments were approved by the local animal care and use committee and comply with current regulations covering animal experimentation in Germany and the EU (European Communities Council Directive 86/609/EEC).

### Morris water maze

To investigate spatial learning we established a non-cued version of the Morris water maze [31] modified for guinea pigs according to [21]. The maze was a circular pool with a diameter of 160 cm, filled to a height of 35 cm with water (temperature 25 ± 1°C). For spatial orientation eight different geometric shapes made of black adhesive film were placed on the inside of the white edge of the pool, a few centimetres above the water surface. The objection of the test was to find a platform 20 cm in diameter made of translucent acrylic glass. The platform was hidden 2 cm below the water surface in the middle of one quadrant of the pool, 30 cm away from the wall.

Tests began at an animals' age of 35 ± 2 days. In the acquisition phase guinea pigs were tested in ten trials over five consecutive days, given two trials per day. Each trial had a maximum duration of 45 s and started by gently placing a guinea pig into the water with its head towards the pool wall on the opposite side of the quadrant where the platform was. During the acquisition phase the position of the platform was fixed. If an animal found the platform within the 45 s, it was left to stay on the platform for 15 s. In cases the animals did not find the platform within 45 s they were placed on the platform manually. This procedure was repeated up to three times per trial if a subject did not stay for at least 15 s on the platform. Between the two trials of each day, all animals were placed back in their home cages to recover and dry for five minutes. The position of the platform was fixed for each individual, but positions were changed between subjects. Five days after the last trial animals were tested in the water maze without the platform for 60 s. In this probe trial the time each subject swam in the formerly right quadrant was examined in order to measure spatial memory. In brief, those animals that developed a spatial association of the platform position are expected to spend significantly more time in the formerly rewarded quadrant while animals without a spatial association will spend equal amounts of time in all four quadrants. On the same day, five minutes after the probe trial the platform was placed in the opposite quadrant and two retention trials were performed in order to measure the ability to generalize the task by re-learning of a new position.

All trials were tracked automatically by a digital tracking system [37] assessing path-length, swimming speed, latency to escape from the water, and the time spent in the correct quadrant. Animals that showed 'floating' behaviour (swimming speed less than 0.5 km/h in five or more of the ten trials in the acquisition phase) were excluded from further analysis.

### Statistical analysis

Graphics presented and statistics carried out were done using the statistical software "R" Version 2.7.2 [38]. Data was checked for normal distribution using Q-Q plots [39] for visual inspection and one-sample Kolmogorov-Smirnov tests to test for deviation from normal distribution. Homogenity of variance was checked using Levene's test for homeogeneity of variance across groups. Weight data were analyzed by a repeated measures ANOVA with domestication and sex as between subject factor and day one and day five of weighing as the repeated measure. For comparison of learning performance between wild and domestic guinea pigs and between sexes, the areas under the learning curves, calculated for each individual from trial two to trial ten, were subjected to a two-way ANOVA with domestication and sex as between subject factors. Bonferroni corrected t-tests were calculated for post-hoc analysis. The time spent in the formerly right quadrant during the probe trial was analyzed by means of one-sample t-tests (testing the deviation from chance-level, i.e., 15 s). Re-learning of a new platform position was tested by non-parametric exact two sample Wilcoxon tests since some of the data could not be transformed to normal distribution. As only a significant decrease of the parameters between the two trials was considered to be meaningful in terms of learning, these tests were conducted one-tailed. All other comparisons were done two-tailed. For all tests a significance-level (α) of 0.05 was selected.

## Results

### Body weight and swim speed

During the testing procedure all animals gained weight from day one of testing to day five. Domestic guinea pigs weighed more than wild cavies and females weighed less than males (repeated measures ANOVA; day: F1,97 = 4.7, p = 0.03; domestication: F1,97 = 213.53, p < 0.001; sex: F1,97 = 35.03, p < 0.001). Additionally, there was a significant interaction of the weight between sex and domestication (F1,97 = 8.6, p = 0.004), reflecting that the sex dimorphism is greater in domestic guinea pigs than in wild cavies.

**Figure 1:**
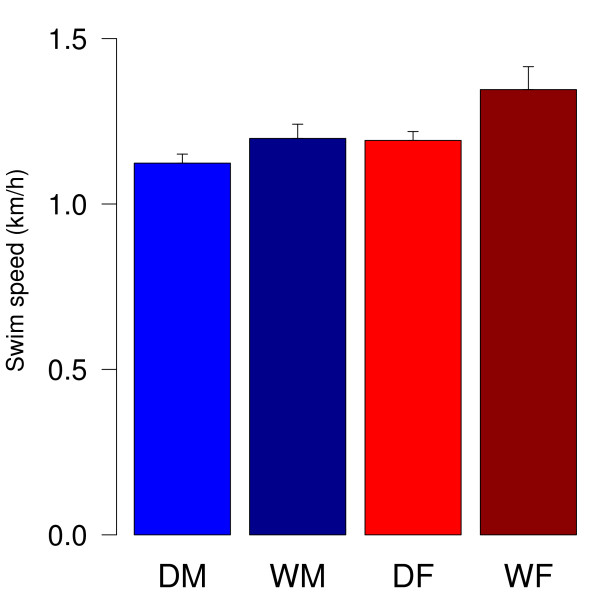
**Swim speed in the Morris water maze task**. Data represent mean (+SEM) speeds of trial one to ten. DM = male domestic guinea pigs (n = 15), DF = female domestic guinea pigs (n = 13), WM = male wild cavies (n = 13), WF = female wild cavies (n = 13). ANOVA revealed significant effects of sex and domestication (sex: F1, 50 = 6.38, p = 0.015; domestication: F1,50 = 5.21, p = 0.027).

Wild cavies and domestic guinea pigs were both observed to be well swimmers using forelegs and hind legs ipsilateral synchronously while swimming. Only one male domestic guinea pig was characterized as being a 'floater' (swimming speed less than 0.5 km/h in seven trials) and excluded from further analysis. Male domestic guinea pigs swam at a speed of 1.12 km/h on average and females at an average speed of 1.19 km/h. In the group of wild cavies average swimming speeds were 1.18 km/h for males and 1.35 km/h for females. Statistical analysis revealed a significant effect of sex with females being faster than males and a significant effect of domestication with wild cavies being significantly faster than domestic guinea pigs (ANOVA; sex: F1,50 = 6.38, p = 0.015; domestication: F1,50 = 5.21, p = 0.027; Fig. 1). There was no interaction effect of sex and domestication (ANOVA; F1,50 = 1.11, p= 0.3). Post hoc analysis did not bring about statistically significant differences between the groups apart from trend levels that do not withstand Bonferroni correction (t-tests; DM vs WM: t(23) = -1.44, p = 0.16; DF vs. WF: t(24) = -2.06, p = 0.0503; DM vs. DF: t(26) = -1.77, p = 0.088; WM vs. WF: t(21) = -1.63, p = 0.12).

### Acquisition phase

**Figure 2:**
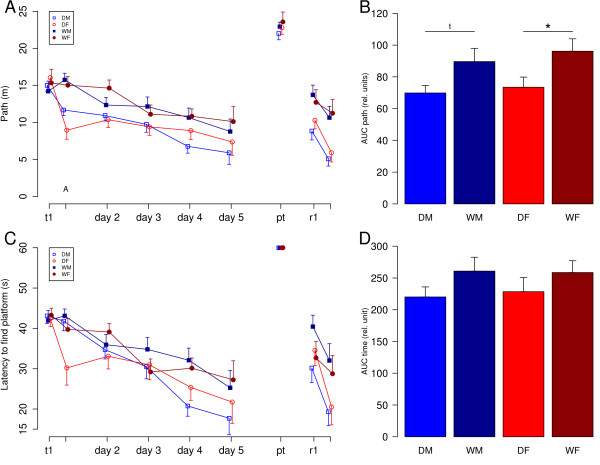
**Learning performance in the Morris water maze task**. DM = male domestic guinea pigs (n = 15), DF = female domestic guinea pigs (n = 13), WM = male wild cavies (n = 13), WF = female wild cavies (n = 13). **A**) Learning curve analyzed by path length. The two trials of the first day are depicted separately. Data for day two to day five are combined values of two trials per day. In the probe trial (pt) the platform was removed. Two retention trials (r1 and r2) were conducted on the same day with the platform being moved to the opposite quadrant of the pool. Data represent means and SEMs. **B**) Comparison of the groups by analysis of the areas under the learning curves (AUC) of the parameter 'path length' calculated for the acquisition phase (trial one, day one to trial ten, day 5). Data represent means + SEM. ANOVA revealed a significant effect of domestication (F1, 47 = 9.32, p < 0.01). Statistical symbols of post hoc analysis are depicted in the figure. * = p < 0.05, t = p < 0.1. **C**) Learning curve analyzed by latency to find the platform. **D**) Comparison of the groups by analysis of the areas under the learning curves (AUC) of the parameter 'latency' calculated for the acquisition phase (trial one, day one to trial ten, day 5). ANOVA revealed effects of domestication as a statistical trend (F1, 47 = 3.02, p = 0.09).

Domestic guinea pigs and wild cavies of both sexes showed the ability to solve the task by finding the hidden platform. During the acquisition phase of all experimental groups, learning curves decreased from trial one to ten regarding both parameters *latency to escape from the water onto the hidden platform *as well as the *path length swum to reach the platform *(Fig. 2). The analysis of main effects of sex indicated no difference in learning behaviour of males and females neither in latency nor in path length (ANOVA; latency: F1,47 = 0.03, p = 0.86; path length: F1,47 = 0.5, p = 0.48). In contrast, ANOVA revealed a main effect of domestication for the parameter *path length*, with domestic guinea pigs covering shorter path lengths than wild cavies indicated by a comparison of the areas under the learning curves (F1,47 = 9.32, p < 0.01). Post hoc t-tests confirmed significant differences between female domestic guinea pigs and female wild cavies (t(24) = -2.25, p = 0.034) and revealed a strong trend for the difference between male domestic guinea pigs and male wild cavies (t(23) = -2.06, p = 0.051). Regarding the parameter *escape latency*, the ANOVA calculated on the areas under the learning curves revealed a statistical trend for a domestication main effect with domestic guinea pigs being faster than wild cavies (F1,47 = 3.02, p = 0.09). There were no interaction effects of sex by domestication (path length: F1,47 = 0.04, p= 0.8; escape latency: F1,47 = 0.07; p = 0.8).

### Probe trial

**Figure 3:**
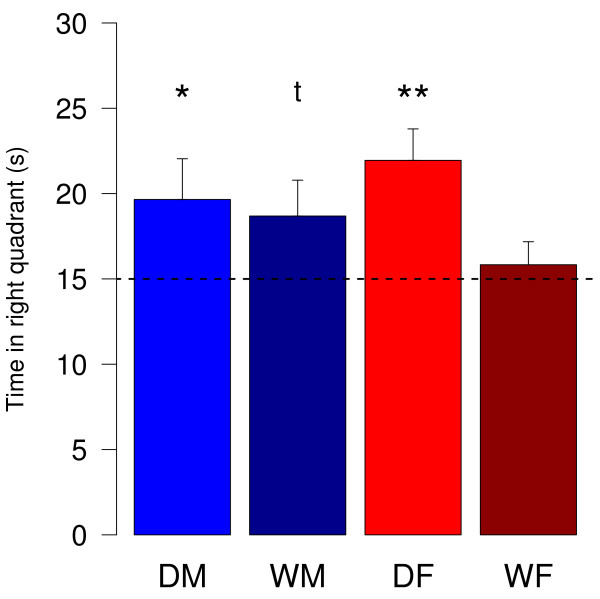
**Time spent in the right quadrant of the Morris water maze**. In the probe trial the platform was removed and animals explored the pool for 60 s. Data represent mean (+SEM) time the animals were recorded to be in the quadrant of the pool where the platform used to be in previous trials. DM = male domestic guinea pigs (n = 15), DF = female domestic guinea pigs (n = 13), WM = male wild cavies (n = 13), WF = female wild cavies (n = 13). Statistics: one-sample t-test testing the deviation from chance-level (dotted line). ** = p < 0.01, * = p < 0.05, t = p < 0.1.

Five days after the last trial of the training phase, a probe trial of 60 s without a platform was conducted. The time the subjects spent in the formerly right quadrant of the pool was measured and analyzed for deviation from chance-level (Fig. 3). Both, female and male domestic guinea pigs spent significantly more than 15 s in the quadrant, where the platform was formerly located (one-sample t-tests: DM: t(14) = 1.95, p = 0.04; DF: t(12) = 3.76, p = 0.001). Male wild cavies differ from chance-level of 15 s by trend (one-sample t-test: t(12) = 1.75, p = 0.053). However, female wild cavies did not spent significantly longer in the quadrant of the former platform location (one-sample t-test: t(12) = 0.62, p = 0.27).

### Retention trial

After the probe trial, two additional trials were conducted with the platform positioned in the opposite quadrant. The performance measured as path length and latency to find the platform advanced between both trials, indicating learning processes. Statistic analysis revealed a significant decrease of the path length between the two trials for all groups but female wild cavies (DM: t(14) = 2.4, p = 0.015; DF: t(12) = 2.997, p = 0.006; WM: t(12) = 1.997, p = 0.035; WF: t(12) = 0.52, p = 0.31). The same was true for the parameter latency to find the platform (exact two sample Wilcoxon tests: DM: W = 82, p = 0.033; DF: W = 54, p = 0.002; WM: W = 32, p = 0.03; WF: W = 34, p = 0.28).

A comparison of the groups in the final trial, revealed a significant effect of domestication with domestic guinea pigs outperforming their wild conspecifics in terms of path length and latency to find the platform (path length: F1,50 = 15.07, p < 0.001; latency: F1,50 = 6.56, p = 0.014). ANOVA did not reveal any significant effects of sex and no significant interactions between sex and domestication.

## Discussion

Wild cavies as well as domestic guinea pigs learned to find a hidden platform in the water maze. Domestic guinea pigs had superior skills in this test compared with wild cavies. Overall our findings indicate that these animals are suitable for investigations of learning and memory. This is in line with previously published results indicating that the water maze is an appropriate task to be conducted with guinea pigs [21,29,30,33,34]. One major problem in many earlier attempts to analyze learning behaviour of guinea pigs was their phlegmatic nature when introduced into novel situations as already described over 100 years ago: "A guinea pig will gnaw for five minutes at a freely swinging door without happening to give it a hard enough push to open it. The gentle swinging of the door back and forth seemed to suggest nothing. (...) Even though extremely hungry the little fellow will get discouraged after finding that all the methods he knows fail to reach the food, and he will sit down in a corner of the cage and remain there."[23]. Obviously, placing the guinea pigs into a water basin seems to do the trick and activates them. Indeed, the advantage of preventing the guinea pigs from freezing behaviour by means of flooding the test apparatus with water has been observed earlier [40,41]. Noteworthy, in our study only one animal could not be analyzed due to floating behaviour, i.e., swimming at a speed less than 0.5 km/h. In many studies analyzing rats or mice in the Morris water maze, the confounding rate of floating behaviour is distinctly higher [42-44]. The overall good health status and the fact, that all animals gained weight during the testing procedure indicated that there is no serious welfare issue associated with the task and our testing protocol. Importantly, the natural habitat of wild cavies typically comprises wet areas such as the banks of a lake and small streams [18,19,45], thus it was reasonable to postulate good swimming skills.

Wild cavies have a higher stress responsiveness compared with domestic guinea pigs [36]. Whereas in a study comparing two strains of rats in a water maze, the strain being more reactive to stress showed more floating behaviour [42], in our study opposite results were found with wild cavies swimming faster than domestic guinea pigs. Noteworthy, swimming speed *per se *does not necessarily indicate a surer performance [46]. In our study, higher swimming speed (especially regarding female wild cavies) was not reflected in advances in solving the task. Contrary, the slower swimming domestic guinea pigs outperformed the faster swimming wild cavies. Moreover, differences in swimming speed affect the parameters *escape latency *rather than the parameter *path length*, i.e., a fast swimming animal has to cover the same minimum path length as a slow swimming one. Regarding the path lengths, wild cavies differed considerably from domestic guinea pigs while this drawback was less pronounced regarding escape latency. Nevertheless, the slopes of the learning curves prove that male and female wild cavies also learned the task. However, the data from the probe trial, where the platform was removed, suggests that other than spatial learning strategies have been used by wild cavies. The higher speed at which wild cavies swam, might have contributed to the success of non-spatial strategies [46]. Given that the mean difference between wild cavies and domestic guinea pigs regarding the time spent in the water maze in the last trial was less than 10 s, it is obvious, that whatever strategy was used by the wild cavies, it was a sufficiently successful one. Thus, in future studies focusing purely on spatial memory of wild cavies, we advice to increase the demands for spatial learning e.g., by using a larger pool. Nevertheless, the fact that wild cavies learned the task, might add some ecological relevance to this paradigm as it indicates that participation in this task does not entirely depend on the animals being domesticated.

It is known for many species that males tend to perform better in spatial memory tasks than females and many different hypotheses have been suggested for explanation. Among those the 'range size hypothesis' is suggested to explain most of the described differences in cognition [22]. In brief, this hypothesis predicts that larger territories, including more landmarks demand more spatial skills. Indeed, the home range size of male wild cavies is about 60% - 90% larger than the home range size of females [18,19]. This difference was not reflected in different learning performances between males and females in our study. However, we suggest to not denouncing this theory based on our results due to the limitations of the applied task rendering the possibility of successfully using non-spatial strategies.

The fact, that we did not find an impairment in learning due to domestication in guinea pigs is in line with some earlier observations. Indeed, many domestic species proved to be as good or even better in solving learning and memory tasks compared with their wild ancestral forms. For example domesticated rats were superior to wild rats [47], domesticated gerbils and wild gerbils born in captivity showed similar performance, while gerbils caught in the wild performed less good [13]. Wild foxes were found to be inferior compared with experimentally domesticated foxes in using human gestures, however, in a control task using non-social cues the wild foxes were found to be more skilled [14]. In the same vein, a comparison between dogs and wolves revealed that domestication improved performance in social cognition in reading human communicative signals [48]. Contrary to this, wolves outperformed dogs, when they were reared with daily interactions with humans [49], although it was suggested that this effect might be due to wolves being more willing to participate in the trials [50]. Be that as it may, overall it can be concluded that differences between domesticated and wild ancestral forms might as well be explained by procedural details in favour of either the domesticated or the wild form. Indeed, the domestication of the guinea pig certainly has brought about a variety of changes in several behavioural domains [35] that might also have affected procedural details of testing spatial memory. Especially the motivation for exploratory behaviour is dramatically reduced in domestic guinea pigs compared with wild cavies [36]. Therefore the use of dry mazes might have revealed even contrary results (see [51] for an example comparing wild and laboratory reared house mice). Temperament, on the other hand, is known to differ between domestic guinea pigs and wild cavies. It is argued, that reduced alertness, nervousness, and sensitivity of the domestic form is causally related to a reduction in the reactivity of the stress axes [36]. Such a reduction in stress reactivity is known to also influence learning and memory processes in animal studies [52,53]. As a consequence, the reduced stress reactivity of domestic guinea pigs along with their overall more relaxed attitude possibly constituted a distinct advantage for domestic guinea pigs in the water maze.

## Conclusion

Overall the results proved that both wild cavies and domestic guinea pigs learned to find a hidden platform in a water maze, although the search strategies leading to success were different. The swimming speed of domestic guinea pigs was slower than that of wild cavies which probably has contributed to these differences. Additionally, wild cavies are more responsive to stress than domestic guinea pigs [36], adding to motivational differences. Thus, guinea pigs' domestication as an artificial selection for human desired traits did not led to a degeneration of cognitive capabilities but rather to an adaptation to a man-made environment that allows solving the task even more efficiently.

## Competing interests

The authors declare that they have no competing interests.

## Authors' contributions

LL, TP, NS, and SK designed the experiment and wrote the manuscript. TP conducted the experiments. LL and TP analyzed the data. All authors read and approved the final version of the manuscript.
